# Supplementary figures and images for: Avian antibodies (IgY) targeting spike glycoprotein of severe acute respiratory syndrome coronavirus 2 (SARS-CoV-2) inhibit receptor binding and viral replication
Source: PLoS One. 2021 May 28;16(5):e0252399. doi: 10.1371/journal.pone.0252399 (PMC8162713; doi:10.1371/journal.pone.0252399)

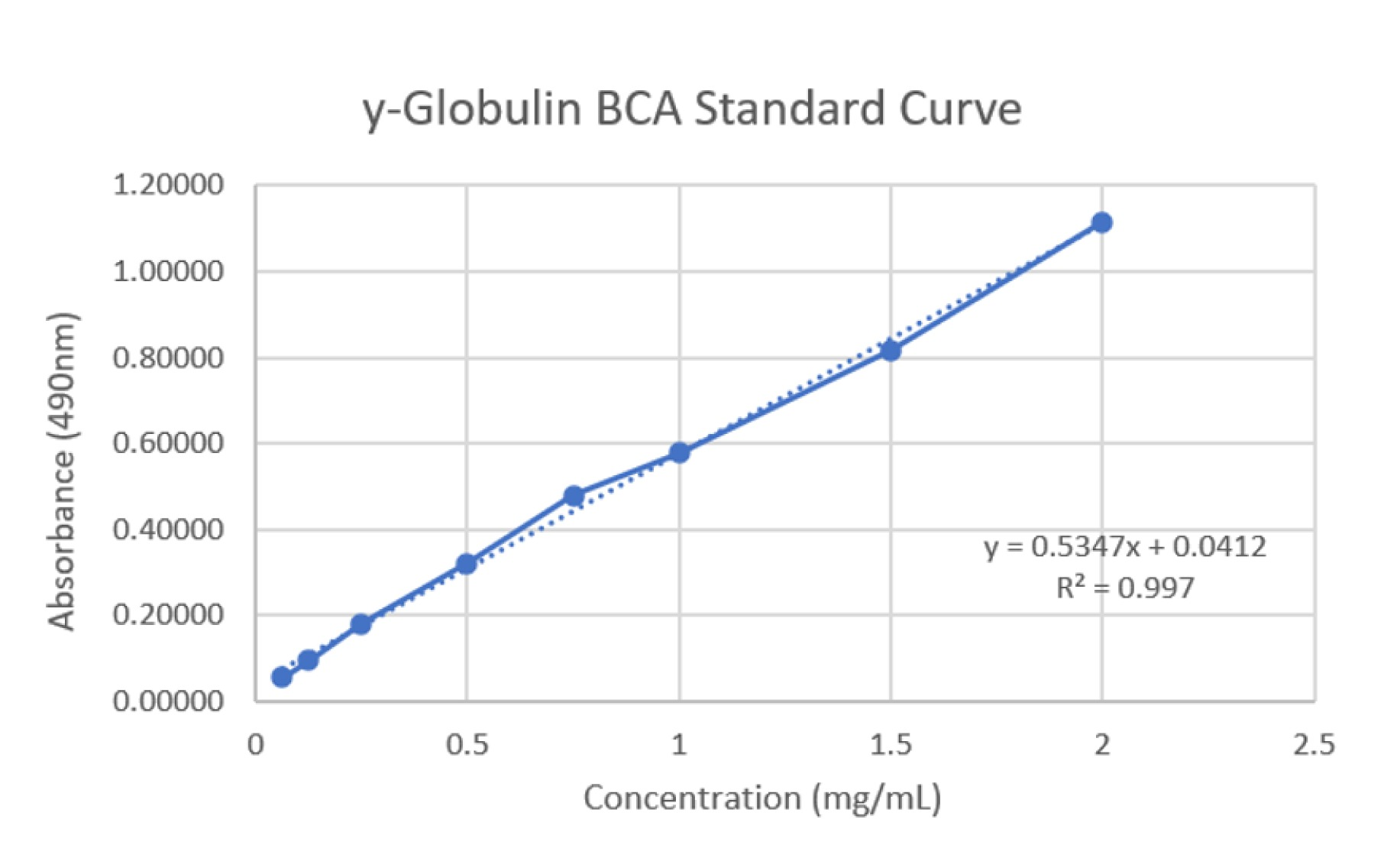

Supplement: S1 Fig — Total protein concentration was determined by BCA method targeting SARS-CoV-2 S1. Curve shows linearity over eight serial dilutions (R2 = 0.99). (TIF) [file pone.0252399.s001.tif]

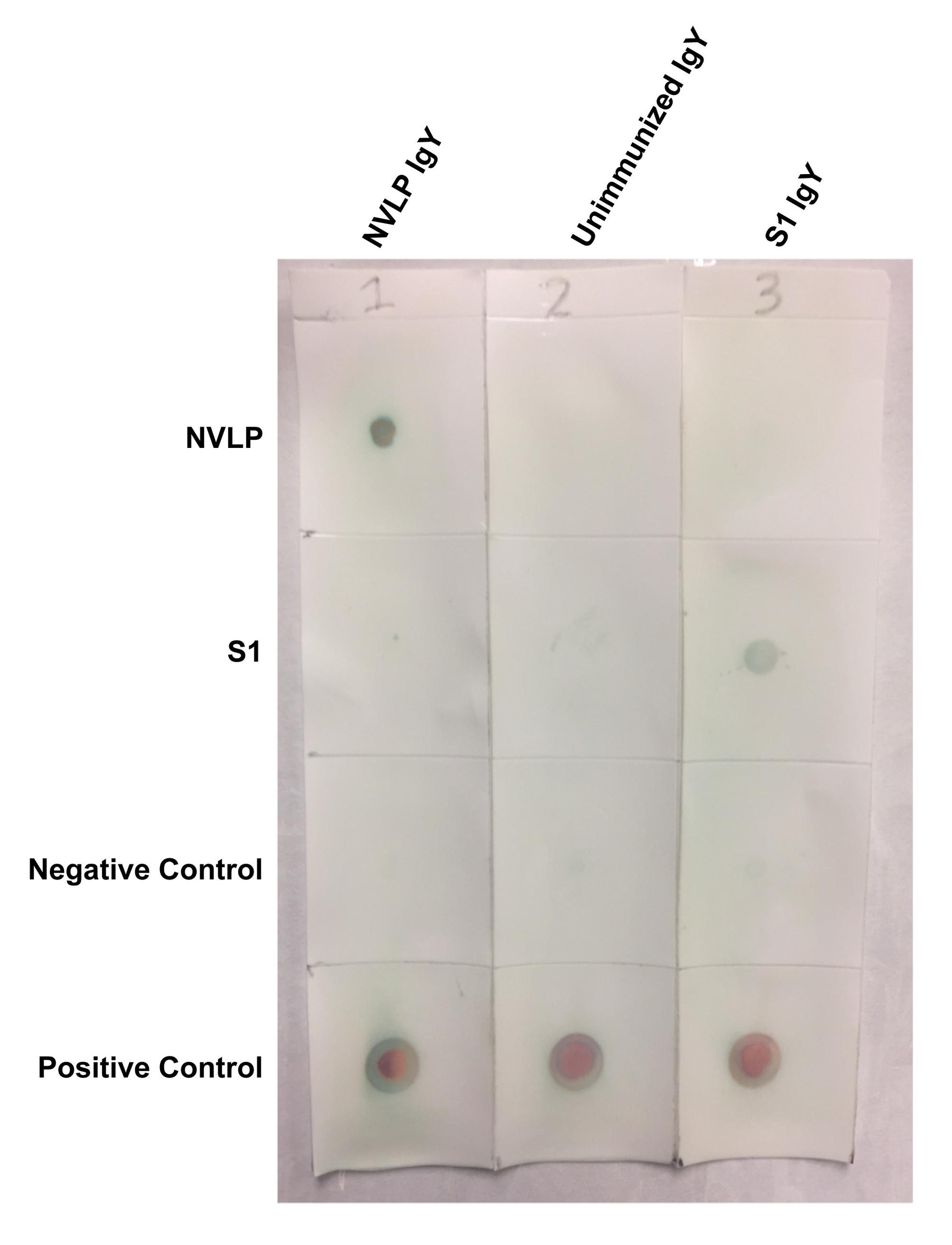

Supplement: S2 Fig — Hens were immunized using norovirus virus-like particles (NVLP), and IgY was prepared as mentioned in the “Materials and methods” section. IgY solutions (NVLP, S1, and unimmunized) were spotted onto a nitrocellulose membrane (Azure Biosystems, Dublin, CA, USA) and allowed to dry at room temperature (RT) for 30 minutes. Each membrane was blocked in tris-buffered saline and Tween 20 (TBST) supplemented with 5% (v/v) skim milk for 1 hr at RT. Membranes were incubated with the appropriate IgY dilutions (1:1,000 anti-S1; 1:2,500 anti-VLP, and Unimmunized IgY) in blocking buffer at RT. After 1 hr, the primary antibody dilutions were aspirated, and each blot was washed three times (5 min) with TBST. Blots were then incubated for 1 hr with a 1:1,000 dilution of Goat anti-chicken HRP-conjugated IgG (ImmunoReagents, Inc., Raleigh, NC, USA) in blocking buffer at RT. Secondary antibody solution was then aspirated, and blots were washed three times (10 min followed by two 5 min washes) with TBST and washed a final time (5 min) with TBS. Color change was observed using TMB chemical substrate (VWR International, Radnor, PA, USA), per manufacturer’s directions. The reaction was quenched using TBS, and images were captured using a standard camera. (TIF) [file pone.0252399.s002.tif]

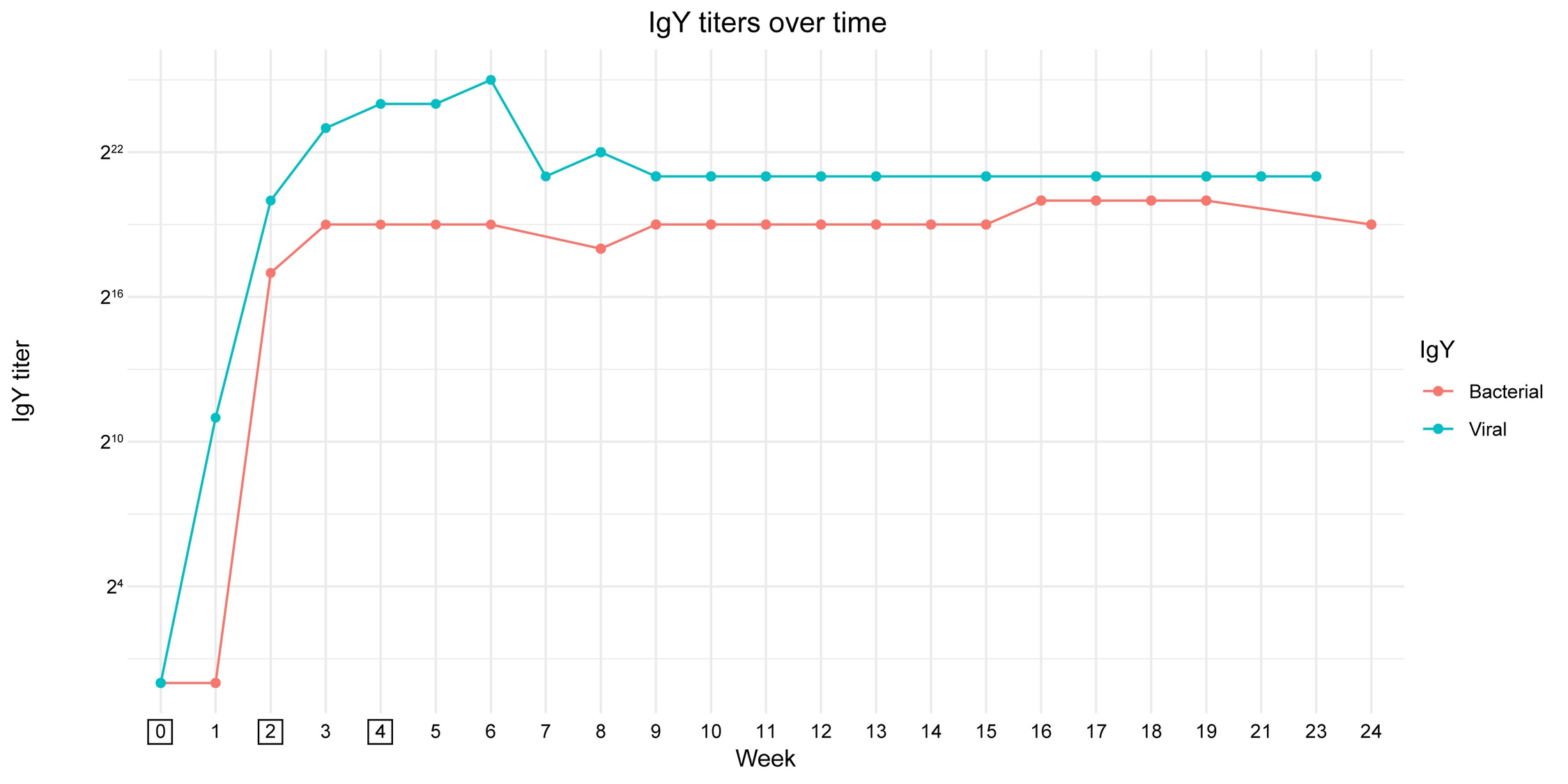

Supplement: S3 Fig — ELISA titers (log2) of IgY targeting norovirus virus-like particles and bacterial multiepitope fusion antigen over 24 weeks are shown as line plots. Viral and bacterial immunizations were carried out intramuscularly, as mentioned in the “Materials and methods” section, at weeks 0, 2, and 4. (TIF) [file pone.0252399.s003.tif]
