# Supplementary material for: Avian antibodies (IgY) targeting spike glycoprotein of severe acute respiratory syndrome coronavirus 2 (SARS-CoV-2) inhibit receptor binding and viral replication
Source: PLoS One. 2021 May 28;16(5):e0252399. doi: 10.1371/journal.pone.0252399 (PMC8162713; doi:10.1371/journal.pone.0252399)
Supplement: S1 File — SARS-CoV-2 inhibitor screen kit method verification was performed following the manufacturer’s specifications [18]. (DOCX) [file pone.0252399.s004.docx]

| Acro Inhibitor Values: S1 RBD | |  |  |
| --- | --- | --- | --- |
|  |  |  |  |
| [Con] (nM) | [Con] log(nM) | OD_450_ | % Activity |
| 0 | N/A | 2.1786667 | 100.0% |
| 0.703125 | -0.15297 | 2.4381667 | 111.9% |
| 1.40625 | 0.148063 | 2.8006667 | 128.5% |
| 2.8125 | 0.449093 | 2.1381667 | 98.1% |
| 5.625 | 0.750123 | 1.6566667 | 76.0% |
| 11.25 | 1.051153 | 1.5806667 | 72.6% |
| 22.5 | 1.352183 | 0.8156667 | 37.4% |
| 45 | 1.653213 | 0.6066667 | 27.8% |
| 90 | 1.954243 | 0.5616667 | 25.8% |
| 180 | 2.255273 | 0.6301667 | 28.9% |

S1 File

Acro SARS-CoV-2 Inhibitor Kit Verification Performance Tables (cf Ref. 18)

| Acro Inhibitor Values: Spike1 Protein | |  |  |
| --- | --- | --- | --- |
|  |  |  |  |
| [Con] (nM) | [Con] log(nM) | OD_450_ | % Activity |
| 0 | N/A | 1.714 | 100.0% |
| 0.703125 | -0.15296746 | 1.6938 | 98.8% |
| 1.40625 | 0.148062535 | 1.5528 | 90.6% |
| 2.8125 | 0.449092531 | 1.4743 | 86.0% |
| 5.625 | 0.750122527 | 1.4483 | 84.5% |
| 11.25 | 1.051152522 | 1.1928 | 69.6% |
| 22.5 | 1.352182518 | 0.9313 | 54.3% |
| 45 | 1.653212514 | 0.6878 | 40.1% |
| 90 | 1.954242509 | 0.4968 | 29.0% |
| 180 | 2.255272505 | 0.3738 | 21.8% |
