# Supplementary material for: Avian antibodies (IgY) targeting spike glycoprotein of severe acute respiratory syndrome coronavirus 2 (SARS-CoV-2) inhibit receptor binding and viral replication
Source: PLoS One. 2021 May 28;16(5):e0252399. doi: 10.1371/journal.pone.0252399 (PMC8162713; doi:10.1371/journal.pone.0252399)
Supplement: S1 Raw image — (PDF) [file pone.0252399.s006.pdf]

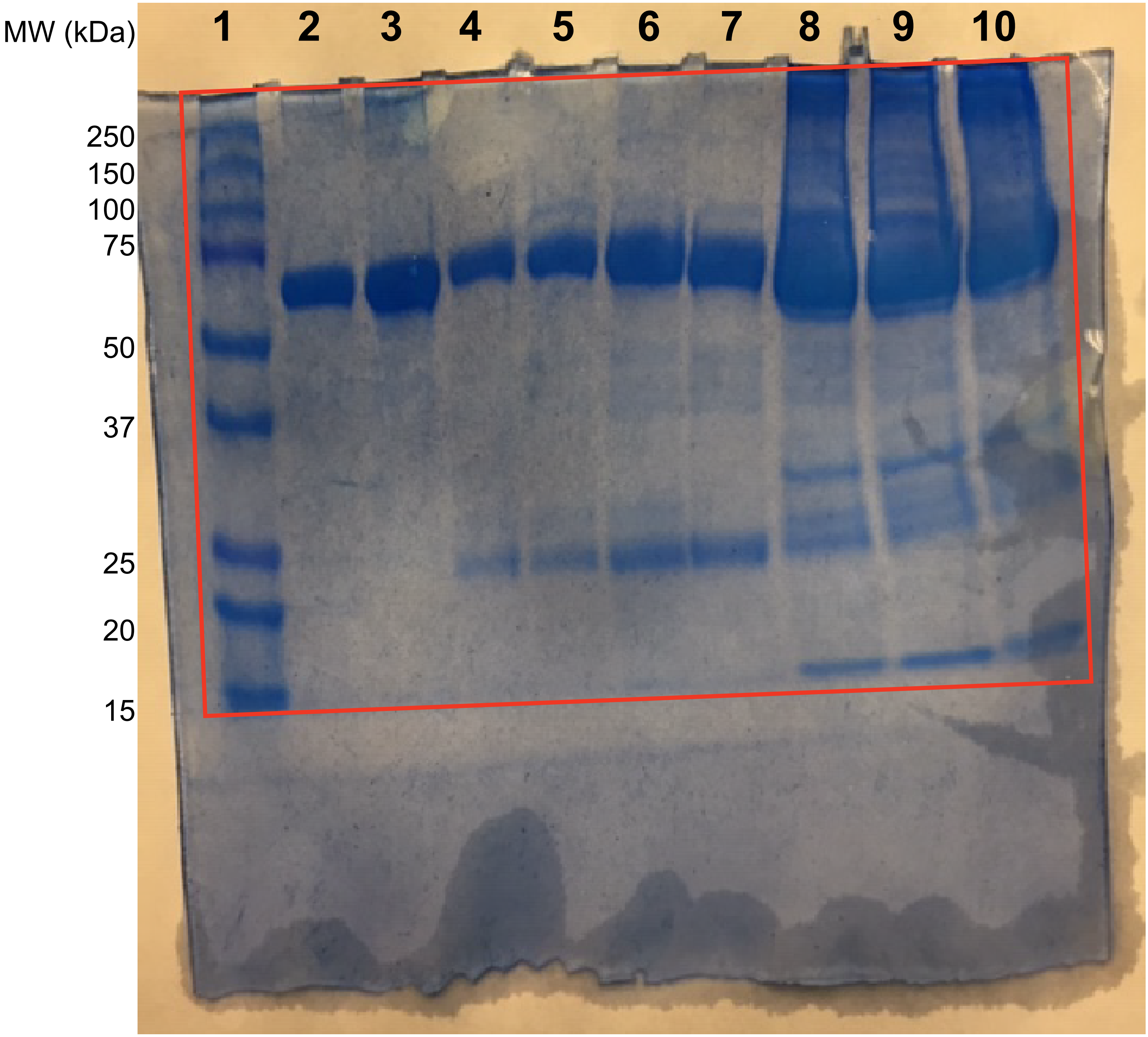

S2 Raw Image. SDS-PAGE analysis of SARS-CoV-2 S1 spike-specific IgY carried out under reducing conditions.

Lane loading pattern: 1, molecular weight marker; 2, BSA standard (1 mg/ml); 3, BSA standard 2 mg/ml; 4, S1 yolk IgY pooled from hen pair (week 0); 5, S1 yolk IgY pooled from hen pair (week 3.5); 6, S1yolk IgY pooled from hen pair (week 4); 7, control yolk IgY pooled from hen pair(week 4; unimunized hens); 8, S1 serum IgY from hen A (week 4); 9, S1 serum IgY from hen B (week 4); 10, control serum IgY pooled from hen pair (week 4; unimunized hens).

Image was captured using an iPhone X (Apple, Cupertino, CA, USA). To create **Fig 2** in main text, the original image was cropped (red box) and the background was adusted for brightness using Adobe Illustrator 2020 v.24.2.1 (Adobe, San Jose, CA, USA).
